# Glomus tumor: revitalizing concepts

**DOI:** 10.1590/0100-3984.2014.0090

**Published:** 2015

**Authors:** Maurício Fabro, Sara Raquel Madalosso Fabro, Bárbara Blaese Klitzke, Gustavo Lopes de Araújo, César Augusto Machado

**Affiliations:** 1 Hospital Santa Catarina de Blumenau, Blumenau, SC, Brazil.

*Dear Editor*, 

A female, caucasian, 22-year-old patient with no comorbidity, complaining of incapacitating crises of subungual pain in her left hallux, strong enough to wake her up in the middle of the night, starting four years ago, with progressive worsening. The symptom was triggered by a physical stimulus such as cold temperature, local pressure, and even wind causing the crises which improved as she immersed her feet into tepid water. The patient had already sought medical assistance several times, receiving multiple, different treatments, including treatment for mycosis, tendinitis and neuritis. Amongst the prescribed pharmaceuticals, she reported the use of analgesic, nonsteroidal anti-inflammatory and corticosteroid drugs, all of them with no response.

**Figure 1:**
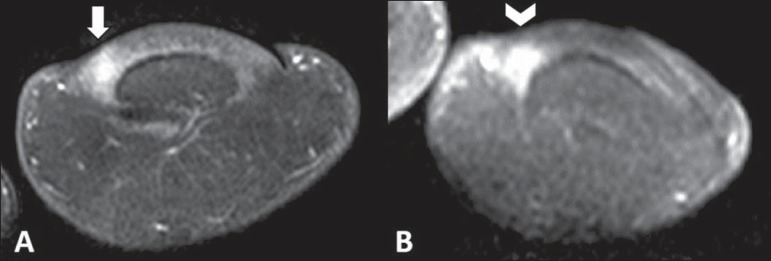
Magnetic resonance imaging – coronal sections, T2-weighted (**A**) and contrast-enhanced T1-weighted (**B**) sequences showing area of hypersignal (arrow) followed by homogeneous radiopharmaceutical uptake (arrowhead).

**Figure 2:**
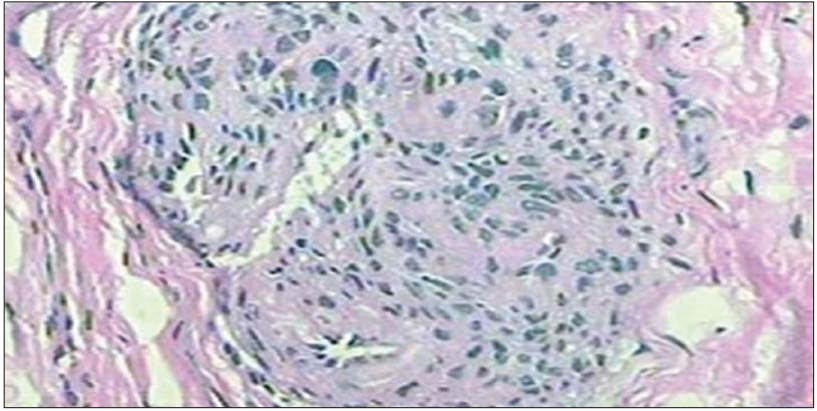
Histological section – hematoxylin-eosin staining showing typical appearance of a glomus tumor.

Both plain radiography and ultrasonography of the first digit of her left foot did not characterize any pathological finding. Magnetic resonance imaging of her left hallux demonstrated the presence of a subungual solid, well-delimited nodule with hyposignal and homogeneous contrast uptake at T1-weighted-image (Figure 1). Such findings suggested the diagnosis of a glomus tumor, confirmed by histopathological analysis of surgical specimen following the surgical removal of the lesion (Figure 2). Complete symptoms resolution was observed after the surgical intervention.

Glomus bodies are arterio-venous shunts present in several parts of the body, with greater concentration in the reticular layer of the dermis, especially located in the digits, palms of the hand and sole of the feet^(1)^. Such structures are responsible for thermoregulation by means of skin blood flow control, being constituted by an afferent arteriole, an anastomotic vessel named Soucquet-Hoyer canal involved by smooth muscle fibers, an afferent vein, nervous fibers and a peripheral capsule^(1,2)^.

Glomus tumors are rare benign lesions characterized by hamartomatous proliferation originating from glomus bodies. Such tumors correspond to 2% of all primary soft part tumors and to 1%-5% of all soft part tumors in the hand^(1,3)^. Approximately 75% of glomus tumors occur in the hand, and 60% of them are subungual (a typical location of such tumors)^(1,4)^. Glomus tumors were first described by William Wood in 1812, as a painful, subcutaneous, slow-growing tumor susceptible to temperature variations. Only later, in 1901, Grosser described the lesion as arteriovenous anastomoses, associating them with the body temperature regulation^(2)^.

Glomus tumors affect young adult individuals, particularly between their fourth and fifth decade of life, being seven times more frequent in women^(5)^, at a mean age of 39^(6)^. Clinically, hyperalgesia is the cardinal symptom of this tumor, and in 90% of cases the triad paroxysmal pain, local hyperalgesia and hypersensitivity to cold temperatures is present^(5,7)^. The symptoms are triggered by unimportant trauma and variations in temperature, improving with immersion into tepid water^(2)^. Physical examination if usually ineffective, but in some cases ungual alterations^(5)^ and bluish nodules^(1)^ may be found, the latter measuring about 3 to 10 mm in diameter^(1,5,8)^.

The diagnosis is based on the patient’s clinical history and physical examination in an attempt to trigger the pain followed by immersion into tepid water to determine improvement. However, clinical criteria present 50%-90% sensitivity and it takes four to seven years after the symptoms onset to have a diagnosis^(5,6)^. Specific tests have been developed to AID in the diagnosis, as follows: a) Love’s test^(1,5)^ – allows for identifying the exact site of the lesion, by applying localized pressure with the end of a paper clip, with 100% sensitivity; b) Hildreth’s test^(1,5)^ – reduction of the pain after inflation of a tourniquet proximally applied to the arm, with 90.5% sensitivity; c) Transillumination – visualization of subungual blue nodules (on the nail bed), with 85.7% sensitivity^(2)^.

As clinical criteria present low sensitivity, imaging methods play a supplementary role in the diagnosis. Plain radiography fails to demonstrate significant findings, and may fail to depict erosion in 14% to 60% of cases^(5,6)^, increased distance between the dorsal aspect of the distal phalanx and the underside of the nail in 25% of cases^(6)^. Ultrasonography can demonstrate a well defined, solid, hypoechogenic and hypervascularized nodule in 83% of cases^(6)^. However, ultrasonography is limited because of the subungual location of most glomus tumors. On the other hand, Magnetic resonance imaging presents almost 1005 sensitivity, demonstrating a solid nodule with hyposignal on T1-weighted images and hypersignal on T2-weighted images, with homogeneous contrast uptake^(6)^. Surgery is the definite treatment^(1,2,5)^, with a recurrence rate of 12% to 24%^(5,6)^. 
